# Retraction: Post-acute (long) COVID-19 quality of life: validation of the German version of (PAC19QoL) instrument

**DOI:** 10.3389/fpubh.2023.1308285

**Published:** 2023-10-31

**Authors:** Frontiers Editorial Office

Following concerns regarding the originality of the article, an investigation was conducted in accordance with Frontiers' policies. It was found that the complaint was valid and that the article should be retracted, because of an unacceptable level of similarity to an article published by Ulbrichtova et al. Validation of the Slovakian Version of the “Post-acute (Long) COVID-19 Quality of Life Instrument” and Pilot Study. Patient Preference and Adherence. 2023. doi: 10.2147/PPA.S404377

The retraction of the article was approved by the Field Chief Editor of Frontiers in Public Health. The authors agree to this retraction.

